# Development of the INpaTiEnt Rehabilitation App Compliance QuesTionnaire [INTERACT]

**DOI:** 10.3390/jpm13121638

**Published:** 2023-11-24

**Authors:** Hassan Tarek Hakam, Felix Mühlensiepen, Mikhail Salzmann, Jonathan Lettner, Roland Becker, Sebastian Kopf, Robert Prill

**Affiliations:** 1 Center of Orthopaedics and Traumatology, University Hospital Brandenburg/Havel, Brandenburg Medical School, Hochstr. 29, 14770 Brandenburg an der Havel, Germany; 2 Faculty of Health Science, Brandenburg Medical School, 14770 Brandenburg an der Havel, Germany; 3 Center of Evidence Based Practice in Brandenburg (EBB), A JBI Affiliated Group, Brandenburg Medical School, 14770 Brandenburg an der Havel, Germany; 4 Center for Health Services Research, Brandenburg Medical School, 15562 Rüdersdorf, Germany

**Keywords:** compliance, physical therapy, in-hospital setting, rehabilitative care, joint replacement, expert review, telerehabilitation

## Abstract

Background: The elements of previously designed questionnaires do not take into consideration the burdens encountered in an inpatient hospital setting. The purpose of this study is to validate elements of a non-compliance survey in an in-hospital setting and discuss aspects of compliance with telerehabilitative physiotherapy in the early postoperative period. Methods: A literature search was conducted to identify elements that prevent patients from performing their prescribed physical therapy exercises. These items were then evaluated by the expert review technique as described by Ikart. Afterwards, the synthesized survey was handed out to patients for the assessment of the quality of its items. Results: The results of the expert review technique identified some conceptual and grammatical problems. This led to the adjustment of some of the elements. The quality of the resulting questionnaire was deemed to be good, as patients were able to fully understand the concepts and answer accordingly. A statistical analysis was conducted to evaluate the responses. Conclusions: The items of this second questionnaire have proven to be reliable to assess the compliance of patients in an in-hospital setting. These items will be added to the cross-culturally adapted items of a previous questionnaire.

## 1. Introduction

Physical therapy has been proven to be an effective treatment modality in patients with varying musculoskeletal conditions. Patients with conditions ranging from lower back pain to postoperative rehabilitation after knee or hip replacement can benefit from physiotherapeutic rehabilitative intervention [1,2,3]. One factor determining the success of rehabilitative measures is the patient’s compliance with those measures [4]. However, non-adherence with physiotherapeutic care seems to be an active issue in orthopaedic patients, preventing them from achieving optimal outcomes [5]. One systematic review [6] examined a multitude of studies aimed at improving a patient’s adherence to physical rehabilitation. Of the examined studies, only two revealed a statistically significant improvement between the intervention group and the comparators. The interventions included a print-out of the exercises in one study [7] and the addition of cognitive behavioral training in the other [8].

The research group at the Center of Orthopaedics and Trauma Surgery is aiming to conduct a high-quality randomized controlled trial to identify the barriers to compliance with physiotherapeutic care in the early postoperative period as delivered by a mobile health application. Therefore, a non-compliance to physiotherapy survey [9] was cross-culturally adapted and validated [10]. However, the elements were deemed insufficient, as they tackled concerns of patients in private physiotherapeutic practice. Thus, a comparative study contrasting inpatient to outpatient physical therapy might facilitate the identification of some non-adherence parameters. However, a systematic review attempting to compare the effectiveness of inpatient with outpatient physiotherapeutic care found that no scientific evidence is in existence concerning the research question at hand [11].

The effectiveness of delivering musculoskeletal physical therapy by the means of telerehabilitation has been proven for a variety of conditions ranging from lower back pain to rehabilitation after knee or hip replacement [12,13]. In fact, telerehabilitation has been hypothesized to have the same effect as outpatient professionally supervised physiotherapy [14]. In light of this assumption, great implications for the healthcare system can be foreseen, as a decreased workload on physiotherapists would be a direct consequence. This would prove to be beneficial in light of the shortage affecting skilled workers in physiotherapeutic practice [15], the risk of transmitting an infection in the age of the pandemic [16], and the ability to reduce socioeconomic costs by implementing telerehabilitation in everyday practice [17]. However, the effect of mobile health applications on compliance has not yet been studied, and a systematic review attempting to evaluate these apps identified several problems with their conception [18]. The design of the application used for the previously mentioned randomized controlled trial will be the subject of another paper.

The purpose of this study is to conceive items for a questionnaire that tackles non-compliance with physical therapy in an inpatient setting using the expert review technique and to put these elements in light of an early postoperative rehabilitative app-guided physical therapy program.

## 2. Materials and Methods

A three-step method was used in accordance with the suggested approach of Ikart [19]. The first step aimed to identify potential non-adherence elements by conducting a literature search and their synthesis into survey items. The second step included pretesting the synthesized elements using the expert review technique. The final step consisted of standard-pretesting the resulting questionnaire using patient feedback. A total number of twenty-eight patients were surveyed.

To evaluate the mobile application-based approach to physical therapy, the German adaption of the System Usability Score was used [20,21]. At the top of each questionnaire, a self-assessment of compliance to physical therapy for the current and past rehabilitative measures was asked for.

**Figure 1:**
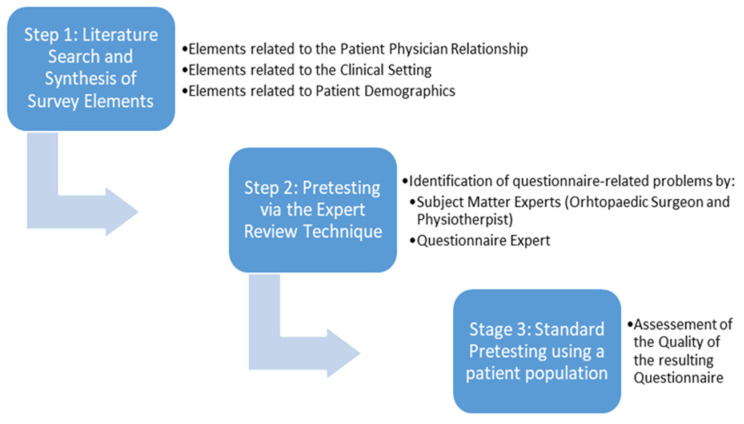
A summary of the steps of the expert review method as applied to the questionnaire at hand.

The methods used for this study are summarized in Figure 1.

### 2.1. Step 1: Literature Search and Synthesis of Survey Elements

In his work, Geisler (1992) identifies physician-related factors that influence their patient’s adherence to a given therapeutic regimen. The empathy the physician shows, his/her skills in motivating patients in taking ownership of their health issues, and the ability to give specific instructions that lead to specific results without over or underestimation were identified as key factors [22].

A cross-sectional study examined the medical staff’s opinions on attendance to physical therapy [23]. The parameters deemed to be applicable for our survey were the patient’s perception about the severity of the disease, the patient’s perception regarding the intensity of the exercise, and the environment in which physical therapy takes place.

Finally, the patient’s demographics correlate with their compliance to physical therapy. Younger patients, male patients, and patients with a higher educational background are more likely to follow health recommendations [24]. As these data are recorded in a standardized manner in any randomized controlled trial, pretesting was not deemed necessary. Another parameter that was identified in this study is the living situation of the corresponding patient. These parameters should be presented at the end of the survey since participants dealing with a questionnaire will have peak interest at the beginning. Afterwards, energy levels of the patient begin to decline, and subsequently, easier questions should be presented in the last section [19]. Patient’s living with their families and receiving support were more likely to be compliant with health recommendations.

### 2.2. Step 2: Pretesting via the Expert Review Technique

The experts were divided into two groups: the survey and questionnaire experts and the substantive or subject-matter experts [25]. The first group was made up of two researchers who are primarily engaged in qualitative research, and the second group was made up of an orthopaedic surgeon and a physiotherapist. All involved experts had many years of academic and research experience.

The experts then evaluated the individual elements using the Questionnaire Appraisal Scheme (QAS) [26]. When an issue was encountered, the appropriate QAS element was mentioned, and an alternative to the element in question was given. The QAS questionnaire will be provided in Appendix A.

### 2.3. Step 3: Standard Pretesting Using a Patient Population

After the questionnaire was reviewed by experts, it was handed to twenty-eight patients for standard pretesting by checking the answers on the written survey. Multiple-choice answers allowed patients to rate the elements from one (completely disagree or false) to five (completely agree or completely right). An extra space was left blank at the end of the questionnaire for comments provided by patients. This step helps in evaluating the quality of the survey. Patients were made aware of their role and asked to provide feedback if any difficulties were encountered.

## 3. Results

### 3.1. Results of Stage 1: Literature Search and Synthesis of Survey Elements

The literature search yielded the items that are presented in the table in Appendix B. Item thirteen was eliminated from the questionnaire, as questioning the support of the prothesis might reveal insecurity towards the support it provides and hinder future execution of exercises.

### 3.2. Results of Stage 2: Pretesting via the Expert Review Technique

The results of the subjective matter expert’s evaluation (orthopaedic surgeon and physiotherapist) as well as the evaluation of the subject-matter expert are presented as tables in Appendix C. The survey items have a designated QAS, a commentary section, and suggestions for improvement. The items are shown in Table 1. The most important modifications included the translation of scientific terms into commonly understood language. “Prothesis” was hence replaced by “artificial joint” and “early postoperative period” was replaced by “first days after the operation”. 

### 3.3. Results of Stage 3: Standard Pretesting Using a Patient Population

The standard pretesting did not reveal any comprehension problems with the questionnaire after the adjustments made in stage 2. Comments provided by patients are presented in Appendix D; the most important comment being that answering the questionnaire depends on the patient’s psychological status as well as the amount of perceived pain.

A simple statistical analysis of the patient’s responses to the individual items are presented in Table 2.

## 4. Discussion

The primary result of this study yielded a non-compliance to the physiotherapy questionnaire adjusted to an inpatient setting. A literature review yielded the synthesis of appropriate items for the survey, an evaluation by subject-matter and questionnaire experts resulted in the refinement of the individual elements, and a pretesting method ensured the quality assessment of the survey. When adding the results of this study to the noncompliance questionnaire provided by “Correlates of exercise compliance in physical therapy”, a comprehensive evaluation of the patient’s motivation to not adhere to physical therapy in a hospital setting can be evaluated. Tackling these issues might lead to an increase in the adherence to recommended therapies and hence ameliorate the outcome of the target population.

The literature search yielded thirteen questions dealing with a variety of aspects of physiotherapy. The physician-specific aspects were discussed in one paper [22] while other studies focused on the environmental aspects affecting the patient’s compliance [23,24]. Intrinsic patient-related factors are discussed in “Correlates of exercise compliance in physiotherapy”. Deeper psychological aspects affecting patient compliance are out of the scope of this questionnaire, and the resulting RCT will exclude patients with psychological disorders that might affect compliance. This aspect, however, is not very well researched and should be considered as a topic for future RCTs dealing with early postoperative rehabilitation. Suggested elements for this type of research can be taken over from studies dealing with non-compliance to pharmaceutical therapy [27]. Hence, this questionnaire only considers the environmental factors that are specific to a clinical setting. Issues like the psychological state and patient expectations are not accounted for in this survey, as they deal with factors that can be controlled in a hospital setting. 

The results of the expert review led to the revision and the adjustment of individual elements. Items were adjusted to imply the patient’s specific situation. QAS items 4 (Vague/Unclear), 7 (Undefined/Vague Term), 15 (Undefined Period), 20 (Complex Estimation), 22 (Undefined Term), and 23 (Vague Term) were used to evaluate survey questions and responses. This led to the adjustments presented in the results of stage 2. Since the patient’s comprehension of the question is key in the evaluation process [28], terms like “postoperative” and “prosthesis” were simplified to “after surgery” and “artificial joint”, respectively. The implementation of precise elements was also tackled, leading to the modification of the terms “early postoperative” and “PT exercises” to the first days after your operation” and to “your prescribed PT exercises”, respectively. German grammatical errors were corrected by the questionnaire expert.

Pretesting on a patient population revealed no comprehension-related issues. Patients primarily used the “Suggestions and Comments” part of the questionnaire to express their views on compliance. The empathy physicians expressed towards the patients’ situations, the motivation physicians provide to patients in taking ownership regarding rehabilitative measures, the presentation of a realistic image concerning the early postoperative period, and the communication of a specific rehabilitative plan all play a substantiative role that affect patients’ adherence to their exercises. Familial support seemed to play the biggest role with regards to compliance with rehabilitative measures. The results of this analysis, however, are not representative for a wide patient population with varying disorders since the evaluation only took place in the confined space of the wards of the orthopaedics and traumatology department and mostly included in-hospital patients treated with reconstruction of the joints.

Generally, the expert review method ensures that multiple dimensions concerning validity are ensured. Face validity was established by searching the literature for commonly perceived problems with compliance and the review of these elements by subject-matter experts and patients [29]. Content validity was ensured by the review of content experts (physiotherapists and orthopaedic surgeons) on the relevance of the presented items and by comments supplemented by patients regarding ambiguity, clarity, and simplicity [30], as the questionnaire addressed the importance of reporting any issue regarding these measures before beginning to answer individual items. Predictive validity is difficult to assess in the realm of future compliance with physical therapy measures, as multiple factors, including the patient’s psychological state, concurrent medical issues, and simple logistic issues like transportation play an immense role [31].

The before-mentioned randomized controlled trial (RCT) will evaluate the effect of an mHealth application on the compliance and the outcome of patients in need of in-hospital rehabilitation. To measure the parameters dealing with the practicality of the application, subjects will be provided with the system usability score (SUS) [32]. Since the SUS is an already validated instrument that aims to evaluate the end-user’s perspectives with respect to the applied system, the research team investigating the use of the mobile rehabilitation application will be implying slightly modified terms to refer to the app. So for example, “system” will be replaced with “app” to facilitate comprehension. These elements will provide the basis on which subsequent modifications targeting the improvement of physiotherapeutic mobile applications will be made.

Finally, the importance of having an active lifestyle and the compliance to physical therapy cannot be understated. New measures to increase the adherence of patients to physiotherapy, specifically, and to an active lifestyle, more generally, must be found. At the Center of Orthopaedics and Trauma Surgery of the University Clinic of Brandenburg, new methods, such as the utilization of a mobile physiotherapeutic reminder application and the use of knee motion sensors, are currently under investigation. While patient-reported compliance to physical therapy is an important measure, the objective assessment of this parameter is of utmost importance, as patient-reported adherence to treatment seldomly reflects the objective reality of the matter.

### 4.1. Limitations

Limitations of this study include the pretesting conducted on patients in German. The use of this questionnaire in English-speaking countries would require the re-evaluation of step 3 using a patient population. As well, this questionnaire did not tackle psychosocial factors such as the patient’s mental health or emotional well-being. This, however, should be taken with caution, as the purpose of this paper is to investigate clinical aspects of compliance in a healthcare facility. Involving the patient’s psychosocial and emotional states is an important aspect that is not in the scope of the current investigation but to which specialists should attend in order to elevate the rate of compliance to the recommended interventions.

### 4.2. Implications for Future Research

This questionnaire can be used in the future to assess environmental factors that lead to non-compliance with physical therapy in Germany and German-speaking countries. The use of this measuring instrument in English should be proceeded by a validation process targeting the affected population, namely, patients undergoing physical rehabilitation after an injury or a surgical procedure. As the relationship between exercise compliance and better outcomes has been proven by previously mentioned studies, this questionnaire will enable orthopaedic surgeons and physiotherapists to investigate and overcome certain barriers dealing with the matter at hand.

## 5. Conclusions

Adherence to physiotherapeutic measures in an inpatient setting can present with some challenges. Physician- and patient-related aspects as well as environmental conditions of the hospital setting play a role in that regard. Additionally, new methods of treatment delivery such as the use of an application might play an immense role in the ever-growing strain on healthcare. A questionnaire was developed in accordance with the “expert review method” to tackle the aspects of compliance in an in-hospital setting. The questionnaire contained aspects of the patient-physician relationship and considerations to the environment in which physical therapy takes place. Additionally, elements of the SUS were added to ensure the quality of the application and its ability to improve the desired outcomes. 

## Figures and Tables

**Table 1:** Represents the elements after modification using the expert review method.

Item Number	Items after Expert Review
1	Was your physician empathetic?
2	How did this affect your adherence to your prescribed physical therapy exercises?
3	Did your physician motivate you to take ownership in ameliorating your situation?
4	How did this affect your adherence to your prescribed physical therapy exercises?
5	Did your physician give you specific recommendations regarding rehabilitation for the first days of in-hospital stay after your surgery?
6	How did this affect your adherence to your prescribed physical therapy exercises?
7	Did your physician give you a realistic assessment regarding the first days after surgery?
8	How did this affect your adherence to your prescribed physical therapy exercises?
9	Did pain hinder your completion of your prescribed physical therapy exercises?
10	Were you confident that your artificial joint will support the execution of the exercises?
11	Was it unpleasant to do the exercises in front of a third party (other patients, medical staff, etc.)?
12	Did your family support you (emotionally/bodily) while performing the exercises?

**Table 2:** Results of the patient’s responses to the individual items. The percentage is presented by the number of responses per total number of participants.

Item Number	1	2	3	4	5
**1**	0	1 (4%)	5 (18%)	4 (14%)	18 (64%)
**2**	0	0	8 (29%)	12 (43%)	7 (25%)
**3**	0	2 (7%)	3 (11%)	9 (32%)	13 (46%)
**4**	2 (7%)	0	4 (14%))	12 (43%)	7 (25%)
**5**	0	3 (11%)	4 (14%))	7 (25%)	10 (36%)
**6**	4 (14%))	0	8 (29%)	9 (32%)	9 (32%)
**7**	1 (4%)	2 (7%)	4 (14%)	10 (36%)	6 (21%)
**8**	7 (25%)	0	11 (39%)	10 (36%)	6 (21%)
**9**	8 (29%)	7 (25%)	3 (11%)	8 (29%)	2 (7%)
**10**	8 (29%)	3 (11%)	14 (50%)	2 (7%)	1 (4%)
**11**	0	2 ()	2 (7%)	13 (46%)	10 (36%)
**12**	1 (4%)	0	0	5 (18%)	21 (75%)

## Data Availability

Additional data are available after request from the corresponding authors.

## Appendix A

Figure representing the evaluation using the Questionnaire Appraisal Scheme (Stage 2):

## Appendix B

Synthesis of the elements after conducting a literature review (results of Stage 1):

**Source**	**Survey Item**
Geisler (1992) [22]	1. Was your physician empathetic and did he stand by your side?
	2. How did this affect your adherence to the PT exercises?
	3. Did your physician motivate you to take responsibility for your situation?
	4. How did this affect your adherence to the PT exercises?
	5. Did your physician give you specific recommendations for your early postoperative rehabilitation?
	6. How did this affect your adherence to the PT exercises?
	7. Did your doctor give you a realistic assessment regarding the early postoperative phase?
	8. How did this affect your adherence to the PT exercises?
Mbada (1970) [23]	9. Did pain hinder you from completing your PT exercises?
	10. Were you confident that your prothesis would support the execution of the exercises?
	11. Was it unpleasant to do the exercises in front of a third party (other patients, medical staff, etc.)?
	12. Were you confident that your direct surroundings (chairs, bed, tables, etc.) would support you while performing the exercises?
Groen (2012) [24]	13. Did your family support you (emotionally/physically) while performing the exercises?

## Appendix C

Results of the Questionnaire Appraisal by the experts:

**Subject-Matter Expert 1: Orthopaedic Surgeon**			
**Item Number**	**QAS Number**	**Comment**	**Suggestions**
1	0	None	None
2	find out	Not very specific	“to your prescribed PT exercises” instead of “the PT exercises”
3	0	None	None
4	0	None	None
5	7 and 15	Some patients might not understand “early postoperative”	Instead use “in the first days after your operation”
6	0	None	None
7	7 and 15	Some patients might not understand “early postoperative”	Instead use “in the first days after your operation”
8	0	None	None
9	4	Patients receive specific programs from the physiotherapists	Use “your prescribed PT exercises instead of “your PT exercises”
10	7	Prosthesis is too scientific	Use “your artificial joint” instead of “prothesis”
11	0	None	None
12	7	Some patients might read this rapidly and understand psychological instead of physical	Use “bodily” instead of “physically”

## Appendix D

Patient’s comments:

Patient 1: “These factors change from day to day. In the first day after the surgery, I would have answered the questionnaire very differently”.

Patient 2: “Familial support is always nice. But having a competent doctor is more important than having an empathetic one”.
